# 457. High Levels of Serum Troponin I Indicate Higher Mortality Risk in Patients with COVID-19

**DOI:** 10.1093/ofid/ofab466.656

**Published:** 2021-12-04

**Authors:** Paula Ramirez, Daniela Parra-Tanoux, Ingrid G Bustos-Moya, Yuli Fuentes, Elsa Daniela Ibañez-Prada, Oriana Narváez - Ramírez, Lina Morales-Cely, Laura A Bravo-Castelo, Salome Gomez-Duque, Enrique Gamboa-Silva, Edar Caceres, Luis F Reyes

**Affiliations:** 1 Universidad de la Sabana, Chía, Colombia, Bogota, Distrito Capital de Bogota, Colombia; 2 Universidad de la Sabana, Bogota, Distrito Capital de Bogota, Colombia; 3 Universidad de La Sabana, Bogota, Distrito Capital de Bogota, Colombia

## Abstract

**Background:**

Up until this day, over 3.5 million fatalities related to coronavirus disease 2019 (COVID-19) have been registered worldwide by the World Health Organization. Healthcare professionals require prognostic tools for COVID-19 patients in order to guide treatment strategies. Elevated troponin levels, a biomarker of cardiac injury, have been detected among patients with COVID-19, hence associating it with cardiac injury. Although several studies have mentioned it, the role of troponin as a prognosis biomarker is unclear. Elevation in troponin levels has been observed in patients with community-acquired pneumonia (CAP). However, its association with mortality is scarcely mentioned in literature. Thus, we sought to determine the utility of serum troponin I levels as a mortality predictor for patients with COVID-19 and CAP.

**Methods:**

A prospective observational study was carried out at Clinica Universidad de La Sabana, Colombia, with patients hospitalized due to CAP and COVID-19. Troponin biomarker was quantified in serum samples using the PATHFAST system within the first 24 hours of hospital admission. Serum concentrations of troponin were compared among study groups. To assess the biomarker′s capacity to predict mortality, ROC curves were used, quantifying their differences through the DeLong′s test.

**Results:**

A total of 88 patients with CAP and 152 with COVID-19 were included in the study. In all cohort the median [IQR] serum concentration of troponin (ng/ml) was higher in those who died (34.2, [9.74-384] vs 5.89, [2.44-27.9] *p< 0.001*). Furthermore, troponin was higher in deceased patients with COVID-19 vs those who survived (77.35 [11.9-346.5] vs. 4.88 [2.10-13.02], *p< 0.001*). However, there was no significant difference between CAP deceased and not deceased patients (18.1 [8.52-398] vs 15.7 [3.75-62.8], *p=0.16*). Although sample size might be a limitation when analyzing these results, the AUC ROC of troponin I to predict mortality was 0.799 for COVID-19 and 0.615 for CAP, the DeLongs test for compared ROC curves was a p= 0.0351.

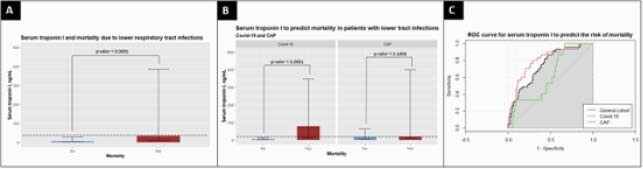

A. Serum troponin I and mortality due to lower respiratory tract infections B. Serum troponin I to predict mortality in patients with lower tract infections C. ROC curve for serum troponin I to predict risk of mortality

**Conclusion:**

Overall, troponin levels were higher among deceased patients. Our findings suggest that high troponin levels are a mortality predictor for patients with COVID-19.

**Disclosures:**

**All Authors**: No reported disclosures

